# Birth of a healthy boy following preimplantation genetic diagnosis for congenital adrenal hyperplasia

**DOI:** 10.5935/1518-0557.20190085

**Published:** 2020

**Authors:** Fakhredin Reihani-Sabet, Poopak Eftekhari-Yazdi, Parnaz Borjian Boroujeni, Javad Roodgar Saffari, Navid Almadani, Shirin Boloori, Mohammad Reza Zamanian

**Affiliations:** 1 Department of Genetics, Reproductive Biomedicine Research Center, Royan Institute for Reproductive Biomedicine, ACECR, Tehran, Iran; 2 Department of Embryology, Reproductive Biomedicine Research Center, Royan Institute for Reproductive Biomedicine, ACECR, Tehran, Iran

**Keywords:** molecular PGD, monogenic disease, preimplantation genetic diagnosis, congenital adrenal hyperplasia (CAH)

## Abstract

Classical 3β-HSD deficiency due to mutations in the *HSD3B2* gene is responsible for a rare form of congenital adrenal hyperplasia (CAH) and is identified by varying degrees of salt wasting. Preimplantation genetic diagnosis (PGD) was performed in a couple carrying mutation c.690 G>A in the *HSD3B2* gene. Four polymorphic short tandem repeat markers closely linked to the *HSD3B2* gene (D1S185, D1S453, D1S514, D1S540) for linkage analysis in conjunction with the direct mutation analysis were used in embryo genotyping. Two CODIS STRs (VWA and THO1) were also used to confirm embryo zygosity and rule out possible contaminations. Finally, *SRY* and *AMYLOGENIN* markers were used for embryo sex determination. PGD was performed by ﬂuorescent multiplex seminested polymerase chain reaction and sequencing. Six embryos were tested and one male carrier embryo was transferred, resulting in the birth of a healthy boy.

## INTRODUCTION

Congenital adrenal hyperplasia (CAH) comprises a group of several autosomal recessive disorders stemmed from the deficiency of one of five enzymes mediating the biochemical steps in steroidogenesis, which include the production of mineralocorticoids, glucocorticoids, or sex steroids from cholesterol by the adrenal glands (Aceto *et al*., 1966; Speiser & White, 2003). Males and females are equally at risk for these disorders (Aceto *et al*., 1966).

In humans, there are two forms of 3 β -hydroxysteroid dehydrogenase enzyme (3β-HSD): type I and type II with 93.5% homology. 3β-HSD enzyme type II (HSD3B2) is responsible for the conversion of Δ5 (delta 5) to Δ4 (delta 4) steroids, and is almost exclusively expressed in the gonads and adrenal cortex. Type I (HSD3B1) is mainly expressed in the mammary gland, placenta, and skin. Both *3β-HSD* genes are located on chromosome 1p13. Enzyme HSD3B2 catalyzes the biosynthesis of progesterone, which is the precursor for aldosterone, and 17α-hydroxyprogesterone, the precursor for cortisol in the adrenal cortex and androstenedione, testosterone, and estrogen in the adrenal cortex and gonads (Rheaume *et al*., 1991; Simard *et al*., 1995). 3β-HSD deficiency is categorized into classical and nonclassical forms (Simard *et al*., 2002).

Classical 3β-HSD deficiency due to mutations in the *HSD3B2* gene is responsible for a rare form of CAH identified by varying degrees of salt wasting (SW). Accordingly, the classical form leads to impaired cortisol synthesis and salt-wasting in its most severe form (Bongiovanni *et al*., 1967; Simard *et al*., 1995). Preimplantation genetic diagnosis (PGD) has been introduced as an alternative to prenatal diagnosis and termination of pregnancy in couples at high risk of transmitting single gene disorders to their offspring.

The goal of PGD is to detect a particular genetic disease on oocytes or embryos obtained through assisted reproductive technologies (ART) before clinical pregnancy is achieved. This is done by selecting and transferring unaffected embryos to the uterus following direct/indirect mutation analysis. In the present case, linkage analysis was performed using polymorphic short tandem repeat (STR) markers closely linked to the mutated *HSD3B2* gene to prepare a further proof of genotyping (indirect analysis) through Sanger sequencing (direct detection) and evaluation for possible allele drop out (ADO). Contamination of exogenous DNA was also evaluated with CODIS-STR markers with different (Fiorentino *et al*., 2010; Gutiérrez-Mateo *et al*., 2009).

This case report emphasizes the successful application of PGD for CAH, which resulted in the birth of a healthy boy. To our knowledge, this is the ﬁrst report of a child born free of CAH after PGD in Iran.

## CASE DESCRIPTION

**Figure 1:**
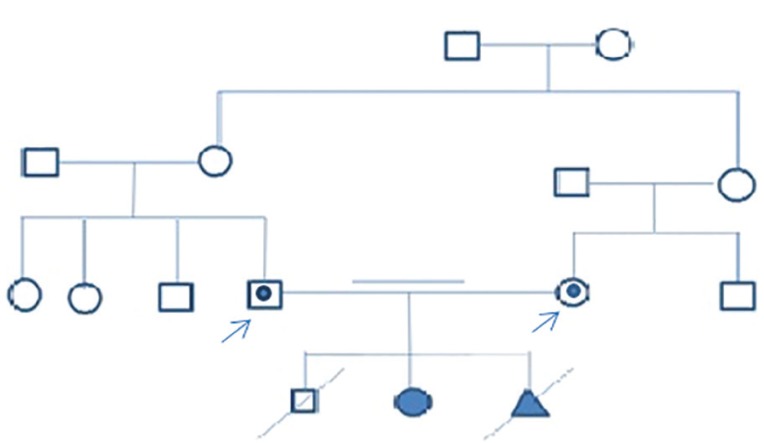
Family tree; the couple (probands) carry the c.690G>A mutation in the *HSD3B2* gene. The index case suffers from CAH

The couple, a 35-year-old woman and a 39-year-old man, was referred to the Royan Institute for genetic counseling. They had had a miscarriage, a dead son to CAH, and a 9-year-old affected girl before coming to our clinic (Fig. 1). Genetic assessment revealed that the couple were heterozygote for the c.690G>A mutation of the *HSD3B2* gene and did not manifest the disease. The couple wished to have an unaffected child and underwent ART with PGD. They also expressed the wish to have a baby boy.

## MATERIAL AND METHODS

Controlled ovarian stimulation was performed via the long protocol of down-regulation with a gonadotropin-releasing hormone agonist and recombinant FSH for 12 days. Seven oocytes were retrieved and six cleaving embryos were available for biopsy on day 3. Blastomeres were lysed for 10 min at 65°C in lysis buffer (dTT 50mM, KOH 200mM) and then analyzed using our in-house PCR protocol.

Preliminary genetic evaluation (Pre-PGD) was performed in order to identify the pattern of inheritance of the affected alleles by determining the presence of the mutation and related patterns of STR markers in the couple’s parents. The PGD protocol was performed according to the ESHRE PGD consortium best practice guidelines for amplification-based PGD (Harton *et al*., 2010). In the first step, 4 ml of peripheral EDTA blood was taken from the couple and their parents. Then, the presence of the mutation was detected by PCR followed by Sanger sequencing. In addition, the pattern of three selected STR markers closely linked to the *HSD3B2* gene was evaluated in the couples and their parents to enable linkage analysis and reveal the associated mutational inheritance. These markers were chosen based on their polymorphism and distance from the *HSD3B2* gene. CODIS STRs and sex selection markers were also designed for further use. In the final step of PGD, semi-nested multiplex PCR was performed using the outer and inner primers listed in Table 1.

**Table 1:** Primer sequences for linked STR markers

Name	Primer F1	Primer F2	Primer R
D1S185	TGCCAGACCCCATAATGGCA	TAATGGCATGAGCCAGTTCT	TCAGGGTCCTCCTAAGAGAA
D1S534	ACATACCATGAGACTTTAGCACA	AGCACATAGCAGGCACTAGC	CGATTGTGCCACTACACAGT
D1S514	AATGCGTGGTCCCAAC	CATTTTTAAACATCCGCACC	GACTCAGACTTCCATCTGGACT

The primer sequences of CODIS STRs used in the test are listed in Table 2. First-round multiplex PCR using the external primers was performed in a total volume of 50µl containing 1.5µl dNTP (10mM), 5µl 10X Buffer (MgCl_2_ 50mmol, 10µl QS, 0.3µl Taq), 10µl Template, 0.2µl (20 pmol) of each primer and 22.5 µl DDW. PCR condition for the first round was 94°C for 3 min, 35 cycles at 94°C for 30'', 60°C for 30'', 72°C for 30'' and 72 °C for 7 min, carried out in an ABI 9700 machine. Direct mutation was analyzed by Sanger sequencing on an ABI 3130 sequencer. The PCR menu was as follows; total volume 20 µl in 5µl 5X Buffer, 1µl Big dye, 0.5µl Primer, 3µl PCR product and 12.5 µl DDW.

**Table 2:** Primer sequences for CODIS STRs

Name	Forward primer	Reverse primer
VWA	GCCCTAGTGGATGATAAGAATAATC	GGACAGATGATAAATACATAGG
THO1	GTGATTCCCATTGGCCTGTTC	ATTCCTGTGGGCTGAAAAGCTC

The PCR program for the sequencing reaction was as follows: 96°C for 1 min, 25 cycles of 96°C 10s, 50°C 5s, 60°C 4 min, and finally incubation at 4°C for 7min. The PCR products of the ﬁrst round were used as templates for the second round of PCR with a total volume of 20 µl, while the conditions for the second round were the same as in the first round. Finally, for direct mutation analysis the ampliﬁed inner products were electrophoresed in an automated genetic analyzer 3130 (Applied Biosystems). The results were analyzed on the Gene Mapper software (Applied Biosystems).

For linkage analysis and sex selection, the amplified PCR products were electrophoresed on 12% acrylamide gel and stained with silver nitrate.

## RESULTS

**Figure 2:**
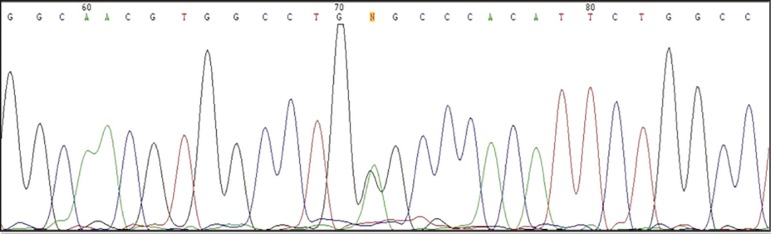
Sanger sequencing results for the c.690 G>A mutation in the *HSD3B2* gene for the transferred embryo

Three STR markers including; D1S185, D1S534, D1S514 and two CODIS STRs of VWA and THO1 were used. SRY and AMYLOGENIN were also used as sex selection markers. The couple had six day-3 embryos. The rate of ADO and contamination were 0%. The biopsy specimen from one of the embryos did not yield amplification, possibly due to the absence of cells. Additional tests confirmed wild type homozygosity in two (healthy), heterozygosity in two (carrier status), and mutant homozygosity (affected status) in one of the embryos (Fig. 2). One heterozygote male embryo was transferred to the mother's uterus and a singleton pregnancy was achieved. Prenatal diagnosis following amniocentesis on the 15^th^ week of gestation confirmed the PGD results and the pregnancy resulted in the birth of a carrier boy.

## DISCUSSION

Preimplantation genetic diagnosis has allowed the identification of various forms of genetic disorders from single embryo cells. PGD offers an authentic reproductive alternative to prenatal diagnosis (PND) and prevents the termination of pregnancies due to fetal affliction resulting from parental genetic disorders. PGD utilizes two strategies to find single gene abnormalities: direct and indirect mutation diagnosis. Although the direct approach is the gold standard in the identification of affected fetuses in PND, the possibility of losing one allele during PCR makes the indirect approach (linkage analysis) a good alternative to improve the validity of PGD results (Alberola *et al*., 2009; Fiorentino *et al*., 2006). For this reason, we used four polymorphic STR markers tied to the mutated gene to provide evidence of ADO (Fiorentino *et al*., 2010; Gutiérrez-Mateo *et al*., 2009).

Steroid 3β-HSD defect is a rare cause of CAH, and is usually detected during the first few months of neonatal life. The diagnosis of this defect is based primarily on increased levels of Δ5 steroid hormones before and after an adrenocorticotropic hormone (ACTH) stimulation test subsequently confirmed by urinalysis (Moisan *et al*., 1999).

Prenatal genetic counseling is recommended for all families affected by CAH. In such an autosomal recessive disorder, one of 6 embryos will be affected when the two parents are carriers (Day *et al*., 1996; Speiser *et al*., 1992). We demonstrated a successful application of blastocyst biopsy, Sanger sequencing, and multiplex PCR for PGD of CAH, in conjunction with sex selection.

In conclusion, multiplex semi-nested PCR was successfully used for the preimplantation genetic diagnosis of CAH, resulting in the birth of a disease-free healthy boy.
